# All You Can Eat: High Performance Capacity and Plasticity in the Common Big-Eared Bat, *Micronycteris microtis* (Chiroptera: Phyllostomidae)

**DOI:** 10.1371/journal.pone.0028584

**Published:** 2011-12-02

**Authors:** Sharlene E. Santana, Inga Geipel, Elizabeth R. Dumont, Margareta B. Kalka, Elisabeth K. V. Kalko

**Affiliations:** 1 Center for Society and Genetics and Department of Ecology and Evolutionary Biology, University of California Los Angeles, Los Angeles, California, United States of America; 2 Institute of Experimental Ecology, University of Ulm, Ulm, Germany; 3 Department of Biology, University of Massachusetts, Amherst, Massachusetts, United States of America; 4 Energy and Resources Group, University of California, Berkeley, California, United States of America; 5 Smithsonian Tropical Research Institute, Balboa, Panama; University of Western Ontario, Canada

## Abstract

Ecological specialization and resource partitioning are expected to be particularly high in the species-rich communities of tropical vertebrates, yet many species have broader ecological niches than expected. In Neotropical ecosystems, Neotropical leaf-nosed bats (Phyllostomidae) are one of the most ecologically and functionally diverse vertebrate clades. Resource partitioning in phyllostomids might be achieved through differences in the ability to find and process food. We selected *Micronycteris microtis*, a very small (5–7 g) animalivorous phyllostomid, to explore whether broad resource use is associated with specific morphological, behavioral and performance traits within the phyllostomid radiation. We documented processing of natural prey and measured bite force in free-ranging *M. microtis* and other sympatric phyllostomids. We found that *M. microtis* had a remarkably broad diet for prey size and hardness. For the first time, we also report the consumption of vertebrates (lizards), which makes *M. microtis* the smallest carnivorous bat reported to date. Compared to other phyllostomids, *M. microtis* had the highest bite force for its size and cranial shape and high performance plasticity. Bite force and cranial shape appear to have evolved rapidly in the *M. microtis* lineage. High performance capacity and high efficiency in finding motionless prey might be key traits that allow *M. microtis*, and perhaps other species, to successfully co-exist with other gleaning bats.

## Introduction

Tropical forests are best known for a high biodiversity that is unmatched by any other terrestrial ecosystem. It has been estimated that one hectare of tropical rainforest can contain 300 species of trees, and may harbor over 41,000 species of arthropods and over 100 species of terrestrial vertebrates [1], [2], [3]. What are the ecomorphological mechanisms that allow species to coexist and partition resources within such diverse communities? Niche theory asserts that sympatric organisms should differ in their use of shared resources as most of these are likely to be limited, and species are predicted to evolve higher degrees of ecological, morphological or behavioral specialization within species-rich communities [4], [5], [6], [7]. Nonetheless, some vertebrate assemblages in the tropics may comprise many species with broad ecological niches, a phenomenon that does not match predictions derived from niche theory. Examples include dendrobatid frogs, some of which are generalist predators that coexist with anuran specialists [8], and Neotropical gleaning bats (Phyllostomidae) [9].

**Figure 1:**
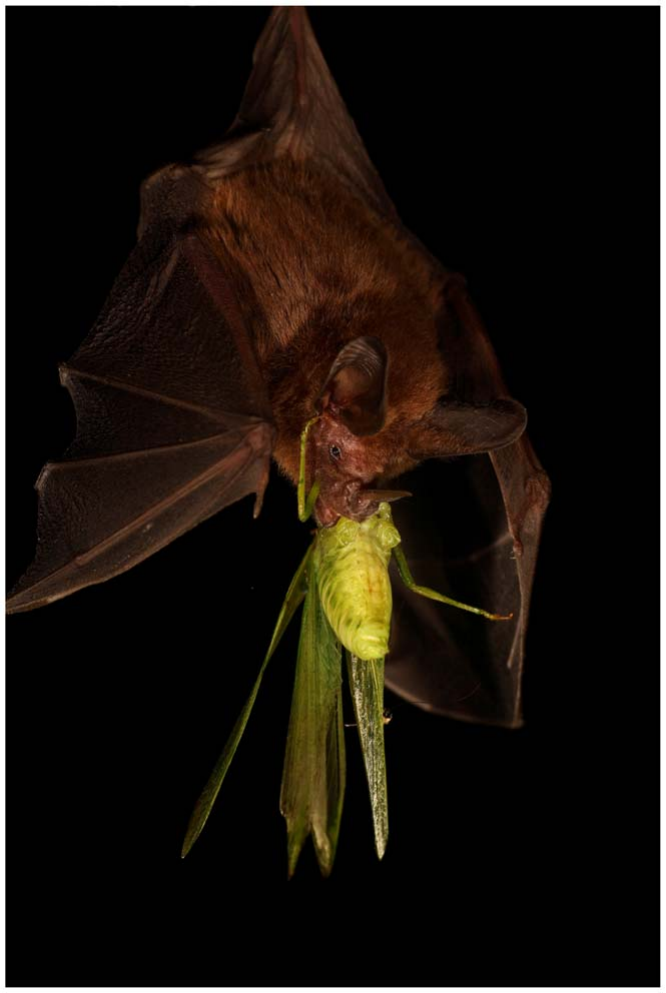
*Micronycteris microtis* consuming a katydid. This image illustrates the relatively large size of some prey items included in the diet of this bat (Photo by Christian Ziegler).

Within Neotropical gleaning bats, the common big-eared bat, *Micronycteris microtis* (Miller, 1898, Phyllostomidae; Figure 1) is a species that takes food from substrates [10] and consumes an unusually wide morphological, ecological and taxonomic spectrum of animal prey when compared to other bats [11], [12]. Prey items consumed by *M. microtis* range from beetles, which are relatively stiff and brittle, to caterpillars, which are ductile and soft [13], [14]. Dietary items span 12 arthropod orders and are usually large with respect to the size of the bats (body mass: 5–7 g, body length of 55–65 mm; Figure 1). Given the relatively broad spectrum of prey consumed by *M. microtis*, this species presents an interesting model to explore the ecomorphological traits that allow for broad resource use within the constraints posed by species-rich tropical communities.

In Neotropical ecosystems, bats are the second most abundant and diverse group of mammals [15], [16], [17]. Therefore, elucidating the mechanisms that might contribute to resource partitioning among bat species would allow for a better understanding of species-rich tropical communities in general. Although it is unclear how bats, and in particular, gleaning species partition resources, current data suggest that niche distinction might be achieved through either differences in the mode and ability to find prey through echolocation and other sensory systems [18], [19], or differences in traits related to food processing, such as bite force and plasticity in feeding behavior [20], [21], [22]. Bite force in bats is strongly predicted by cranial morphology [23], [24] and is a determinant of the prey that are included in the bats’ diet [20]. Recent studies also suggest that most animal-eating species exhibit low plasticity in this performance trait, that is, these bats do not switch their biting behavior as much as frugivores do in response to prey hardness [21]. Insectivores mainly process prey using their molars, and the structure of these teeth is closely associated with their ability to break down prey [25].

Although the interplay among morphology, performance and behavior seem to have an important role in niche partitioning among bat species, there have been at least two main obstacles to understanding the connection between these factors and resource use. First, although we have a general idea about the diets of many species, there are still insufficient diet data for most. Second, it is generally difficult to obtain behavioral information for bats feeding on a wide spectrum of prey. Our purpose was to take advantage of a unique opportunity to quantify the feeding behavior of *M. microtis* to enable a broad understanding of the ecomorphological factors underlying resource partitioning among Neotropical gleaning bats.

Given the relatively broad spectrum of prey consumed by *M. microtis*, we used this species as a model to explore if and how broad resource use is associated with characteristics of cranial morphology, feeding behavior and bite performance. We hypothesized that cranial and behavioral traits related to the generation and modulation of bite force are important ecomorphological factors contributing to *M. microtis*’ broad resource use. We predicted that *M. microtis* has evolved a cranial morphology that allows it to achieve relatively high bite forces and levels of performance plasticity (i.e. the ability to modulate bite force through behavioral changes in response to prey type) when compared to closely related and sympatric animal-eating bats who consume a more limited prey spectrum. We expected *M. microtis* to use its molars predominantly during feeding, but to increase bite force through changes in biting behavior when switching from soft to hard prey, and to vary its biting behavior during the consumption of individual prey items. High performance capacity and behavioral plasticity in *M. microtis* would allow higher flexibility in the use of resources and could be key factors promoting its successful coexistence with other gleaning bats.

## Methods

### Feeding behavior

We filmed processing of native prey captured by *Micronycteris microtis* on Barro Colorado Island (BCI), Panama. Observations were made at feeding roosts located under the external stairs of three residential buildings of the field station, about 2 m from the forest edge. The roosts have been consistently used by *M. microtis* since 1991. A colony of 4–8 (rarely 10–12) individuals used one or two of the roosts every night for social interactions, resting, and feeding. Video recordings were made on 80 nights between 2002 and 2009, usually encompassing several hours of videotaping per night. We placed digital cameras (Type VK-121/IR or Sony Nightshot) on a tripod about 1.2 m from the roost. For most of the recordings, infrared-panels equipped with 32 LEDs (TS AlGaAS infrared 875 nm, 5 mm Ø, HSDL-4230) were used as light sources. To zoom in on prey consumed, we adjusted the camera lens (Eneo DC-Motorzoom Lens EC-Series, F1, 8/8–80 mm) with a remote control. Presence of the camera, which was set outside the main flyways of the animals, and the operation of the motor-zoom did not noticeably affect bat behavior.

Videos were analyzed to describe the types and sizes of prey items, and the feeding behaviors used by the bats. We selected sample of videos in which prey items were easily discernible and identified to order or family level. Prey size (length and minimum width) was estimated by using 34 mm as average forearm length of *M. microtis* as a reference scale. Size measurements of prey were made using ImageJ (National Institutes of Health, Bethesda MD, USA). To further characterize prey items, we collected insects in BCI that were of similar sizes and types as those consumed by bats. We measured the hardness of these in puncture resistance tests performed using a flat-ended needle (1 mm in diameter) attached to a force transducer (see below).

To characterize the feeding behavior of bats and its variation across prey types, we watched video recordings at 1/4 of speed using VLC Media Player (v. 1.0.5 Goldeneye, VideoLAN Team). One observer (SES) counted the total number of bites bats took to eat each individual prey item and categorized each bite as one of four types: shallow bilateral, shallow unilateral, deep bilateral, and deep unilateral [21], [22]. Shallow bites engage the canine and incisor teeth, while deep bites engage the premolar and molar teeth. Unilateral bites use either the left or right tooth row, and bilateral bites simultaneously use both left and right tooth rows. We have demonstrated that bats can use these different bite types to modulate bite force during feeding [21], [22], and that the total number of bites is positively correlated with insect breakdown [25]. Percentages of each bite type and total number of bites were calculated for each prey item and averaged across items of the same taxonomic type (e.g., beetles, cicadas, dragonflies, caterpillars). To assess the behavioral plasticity of *M. microtis* within feeding sequences on prey items of the same type, we identified 20 of the bites as “start bites” (when <10% of the prey was eaten), “middle bites” (when about 50% of the prey was eaten), and “end bites” (when >80% of the prey was eaten) and used this variable as a factor in subsequent analyses (see below).

### Bite force

Adult males and non-pregnant, non-lactating females of *Micronycteris microtis* were captured at roosts on BCI in mist nets. We measured the bats’ bite force (in Newtons) using a piezoelectric force transducer (Kistler, type 9203, range ±500 N, accuracy 0.01 – 0.1 N; Amherst, NY, USA), attached to a handheld charge amplifier (Kistler, type 5995) and mounted between two bite plates [26]. The tips of the bite plates were covered with medical tape to protect the bats’ teeth from damage and to provide a non-skid surface. We adjusted the distance between the bite plates for each individual bat to accommodate a gape angle of about 30 degrees because variation in gape angle might affect measurements of bite force [27]. Bats were usually eager to bite the transducer, or were stimulated to do so by gentle taps at the sides of their mouth. For each bat, we recorded at least five bite force measurements at each of the the four bite positions described above and selected the maximum values per bite position to calculate means. Once bite force measurements were completed, we took standard body measurements (body mass, length of forearm, length, width and height of head). All procedures were approved by Institutional Animal Care and Use Committees at the Smithsonian Tropical Research Institute (protocol # 2008-11-06-24-08) and the University of Massachusetts (protocol # 26-10-06).

### Cranial morphometrics

Ten linear measurements describing skull form were collected from 284 museum specimens, including *M. microtis* (n  =  6) and 67 other phyllostomid species (a subset of the dataset in Dumont et al. in press). Measurements were adjusted for size using log geometric means [28], and variation was summarized by applying a Principal component (PC) analysis to the correlation matrix and a Varimax rotation. This allowed us to construct statistically independent vectors that summarized size-adjusted morphological variation. Out of these vectors, PC1 described a trend of increasing skull length, reduction of the coronoid process, and decreasing skull and condyle height, and was the best predictor of variation in size-adjusted bite forces (i.e. residual of bite force from a regression against head height; Dumont et al. in press).

### Statistical and phylogenetic analyses

We used three approaches to examine the behavioral variation across and within prey types consumed by *M. microtis*. First, we investigated the variation in biting behavior across prey types using a three-way Analysis of Variance (ANOVA). This analysis evaluated the effect of ‘prey type’ (Table 1) on the percentage of each ‘bite type’ (shallow bilateral, deep unilateral, and deep bilateral bites, all arcsin-transformed to ensure linearity). Since the four ‘bite types’ were expressed as percentages they were not independent from one another, thus we deleted one of the ‘bite type’ categories (shallow unilateral) prior to analyses. This allowed us to test for significance of each factor and to generate accurate error terms. Deleting shallow unilateral bites did not affect the results of the ANOVA, as information about this bite type was reflected in the values of the three remaining categories. Second, we investigated the impact of prey type on the total number of bites by using an ANCOVA. In this case, prey length was designated as a covariate, given that total number of bites was significantly correlated with this size measure (R^2^ =  0.353, P  =  0.01). Third, we investigated behavioral variation within feeding sequences by using a three-way ANOVA to test for the impact of ‘sequence point’ (start, middle, end) on the proportion of each ‘bite type’.

**Table 1:** Characteristics of the prey items and feeding behavior of *Micronycteris microtis*.

Prey type	N	Prey length (mm)	Prey width (mm)	Prey hardness (N)	% Shallow bilateral	% Deep unilateral	% Deep bilateral	Number of bites per prey item	Total time per prey item (sec)
Beetles	24	11.86±2.61	4.79±1.75	2.16±0.68	0	70.62±37.39	29.37±37.39	791.24±298.51	177.87±87.11
Caterpillars	45	29.39±9.54	4.13±1.08	–	0	87.00±25.36	10.78±21.68	988.52±560.02	220.40±153.64
Cicadas	2	11.68±0.96	4.25±0.81	1.46±0.42	0	59.17±49.03	7.50±18.37	289.00±1.41	64.50±0.70
Dragonflies	30	35.59±5.42	2.36±0.48	2.36	0	83.83±31.72	9.50±22.21	857.32±354.83	179.30±±69.46
Hymenoptera	9	9.34±1.64	1.20±0.48	–	0	64.44±48.76	24.44±43.33	269.22±34.76	59.00±10.44
Katydids	45	17.49±4.21	3.95±0.92	1.89±0.39	1.67±11.18	84.22±30.98	9.67±22.79	1048.76±696.36	241.67±186.46
Lizards	1	39.25	3.36	–	0	100	0	980	210
Moths	30	17.71±3.99	4.55±1.52	–	0.17±0.91	73.67±32.90	26.17±33.02	682.81±462.32	155.50±112.41
Neuropterans	1	40.13	1.87	–	0	100	0	544.29	127
Roaches	30	17.17±6.39	3.10±0.59	0.46±0.335	0	79.17±37.46	15.83±28.58	1062.20±1354.80	198.40±241.34
Spiders	15	13.32±2.53	3.412±0.78	–	0	67.33±42.58	32.67±42.58	607.84±216.43	146.60±63.61

Shallow unilateral bites are not indicated as these were very rarely used. Sample sizes for hardness of prey items are as follows: beetles: 139; cicadas: 2; dragonflies: 1; katydids: 29; roaches: 2; stick insects: 3. *M. microtis* maximum bite force is 8.25±1.5 N.

To compare the ability of *M. microtis* to modulate its feeding behavior and performance with respect to other phyllostomid species, we compared measures of behavioral and performance plasticity published by Santana and Dumont [21]. These measures reflect the ability of bats to modulate bite performance through behavior when switching from soft (crickets) to hard prey items (beetles). To calculate behavioral plasticity, we added the absolute values of the differences in percentages of each bite type when bats fed on soft vs. hard prey. To calculate performance plasticity, we first calculated “behaviorally adjusted” bite forces by multiplying the percentage of each bite type by the maximum bite force measured on *M. microtis* at that bite position. We then calculated the absolute value of the difference between these behaviorally adjusted bite forces on soft and hard items. This value was expressed as a percentage of the bat’s maximum bite force to account for size differences among species.

Changes in the tempo of evolution in cranial morphology, size-adjusted bite force and performance plasticity were investigated by reconstructing the evolutionary rate in the *M. microtis* lineage and comparing it to the average evolutionary rate across all the species in the dataset. Analyses were run using Dumont et al.’s [in press] species-level phyllostomid phylogeny and following procedures outlined by McPeek [29]. These involve (1) pruning the *M. microtis* branch from the phylogeny, (2) computing the average evolutionary rate across the lineage, (3) grafting the pruned branch onto the phylogeny, (4) calculating the ancestral state for the pruned node, and (5) computing the evolutionary change along the branch leading to *M. microtis*. The rates of character evolution along the branch leading to *M. microtis* were compared to the average rate across phyllostomids using two-tailed t-tests. Analyses were conducted in R 2.11.1 (R Development Core Team, 211) using custom-written scripts and functions from the APE [30] and GEIGER [31] packages.

## Results

### Dietary composition

We quantified behavior for 247 feeding events corresponding to ten arthropod orders representative of the diet of *Micronycteris microtis* (Table 1). Prey items varied in length (range: 7.99 to 91.79 mm) and minimum width (range: 0.88 to 8.15 mm; 1.74 to 8.15 mm, excluding wasps). Hardness measured for prey items within these size ranges were consistently below *M. microtis*’ bite force (Table 1).

**Figure 2:**
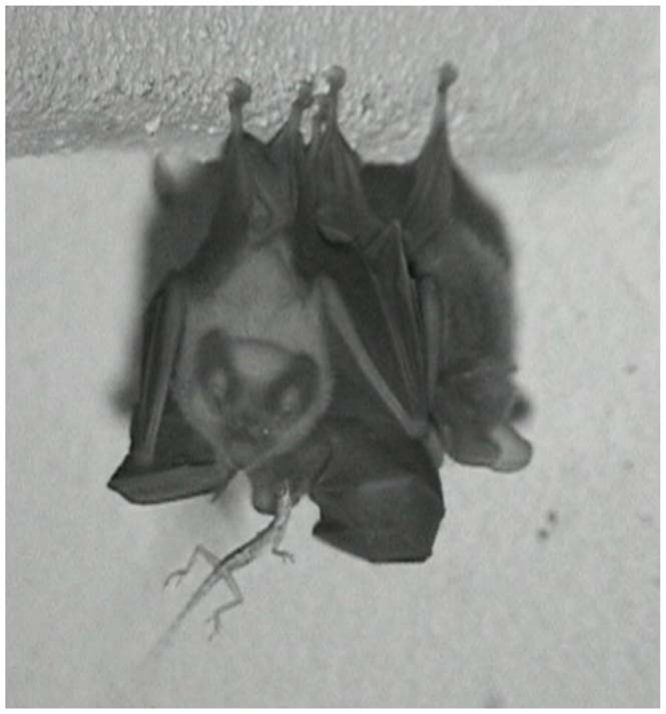
Video snapshot of a group of three *Micronycteris microtis*, as observed in the roost in Barro Colorado Island, Panama. The individual in the center on the back of the group is eating a lizard.

Along with prey items previously reported in the diet of *M. microtis* [12], we recorded for the first time the consumption of vertebrate prey in this genus. Throughout our study, we recorded the consumption of three lizards, apparently small anoles (Figure 2). Out of these three events, image quality only allowed us to quantify bite types from one feeding sequence.

### Feeding behavior

Bats generally ate arthropods by repeatedly biting and crushing the prey’s head, or cephalothorax in the case of spiders, and then biting and discarding the wings, antennae and/or legs. Bats mostly used their premolar and molar teeth for the latter task, biting with one or both sides of the jaw (Figure 1). Once the prey’s head had been consumed and appendices had been discarded, bats consumed the thorax and abdomen biting with their molars and premolars and rotating the prey from one side of the jaw to the other. When eating phytophagous and detritivorous prey (caterpillars, beetles, crickets, katydids, phasmids, cockroaches), bats selectively excluded parts of the intestines by dropping part of the abdomen, or separating it from the exoskeleton by side-to-side head movements [12]. Bats ate lizards in a similar fashion as they did arthropods, except that legs were also eaten along with the whole body. Bats started eating the lizard at the head (Figure 2), where they applied multiple molar bites. They continued to consume the lizard by chewing it with the molars using one side of the jaw, a behavior that continued throughout the consumption of the whole of the lizard. Apparently, lizards were eaten completely; the tail was not dropped.

As expected, *M. microtis* used molar bites predominantly to eat all prey types (Table 1). Nonetheless, the bats changed their feeding behavior significantly with prey type (Table 2). On one hand, they switched between bite types, as demonstrated by the significant ‘prey type x bite type’ interaction term on our ANOVA model (Table 2a). On the other hand, they also used different numbers of bites per prey item across different prey types, even when prey size was accounted for (Table 2b). When consuming individual prey items, bats seemed to vary the proportion of molar bite types used within a feeding sequence (deep unilateral bites: MS  =  15,551.48, F  =  8.97, P  =  0.001; deep bilateral bites: MS  =  9,537.87, F  =  4.24, P  =  0.025).

**Table 2:** Results from Analyses of Variance testing the effect of type of prey on the feeding behavior of *Micronycteris microtis*, (a) Effect of ‘prey type’ (from Table 1) on the percentage of bite types (shallow unilateral, shallow bilateral, deep unilateral, deep bilateral), and (b) Effect of ‘prey type’ (from Table 1) on the total number of bites used to eat a prey item, with prey length as a covariate.

Source	*SS*	*df*	*MS*	*F*	*P*
(a) Response: percentage of bite types					
Prey type	1,194.11	9	132.68	0.713	0.697
Bite type	177,788.42	2	88,894.21	477.79	**< 0.0001**
Prey type × Bite type	10374.76	18	576.38	3.09	**< 0.0001**
(b) Response: total number of bites					
Prey type	0.817	9	0.091	2.735	**0.009**
Prey length	0.051	1	0.051	1.522	0.222
Prey type × Prey Length	0.938	9	0.104	3.137	**0.003**

### Bite performance, plasticity and evolution

**Figure 3:**
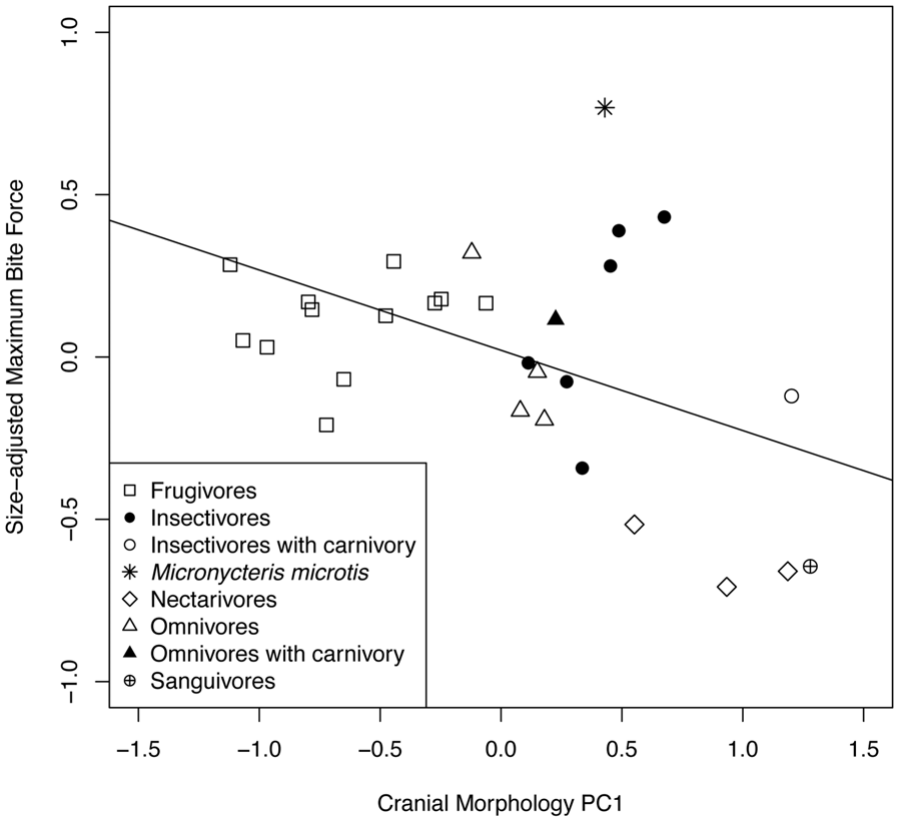
Regression of size-adjusted bite force on cranial morphology for *Micronycteris microtis* and other phyllostomids. Principal Component (PC) 1 was derived from ten linear measurements from dry skulls of M. microtis (N  =  6) and 29 phyllostomid species. PC1 explained most of the variation in bite force (R^2^ = −0.247, F_1,30_  =  10.63, P  =  0.0029). Bite force data for other phyllostomids from Santana & Dumont [21].

We obtained bite force measurements from 17 individual *M. microtis* (8 males, 9 females). Maximum bite force was 8.25±1.519 N (shallow unilateral: 6.91±2.5 N, shallow bilateral; 7.36±1.7 N, deep unilateral: 7.77±1.6 N, deep bilateral: 8.25±1.5 N). When maximum bite force was corrected for size and plotted against cranial morphology, *M. microtis* had the highest positive residual of any species tested (Figure 3). The rates of evolution of size-adjusted bite force and cranial morphology were significantly higher in the *M. microtis* lineage than in the rest of the phyllostomid lineage (bite force: 0.565 vs. 0.029±0.035; t  =  16.836, df  =  35, P  =  1.169 exp-18; PC1: 0.183 vs. 0.064±0.072, t  =  3.424, df  =  63, P  =  0.0005).

**Figure 4:**
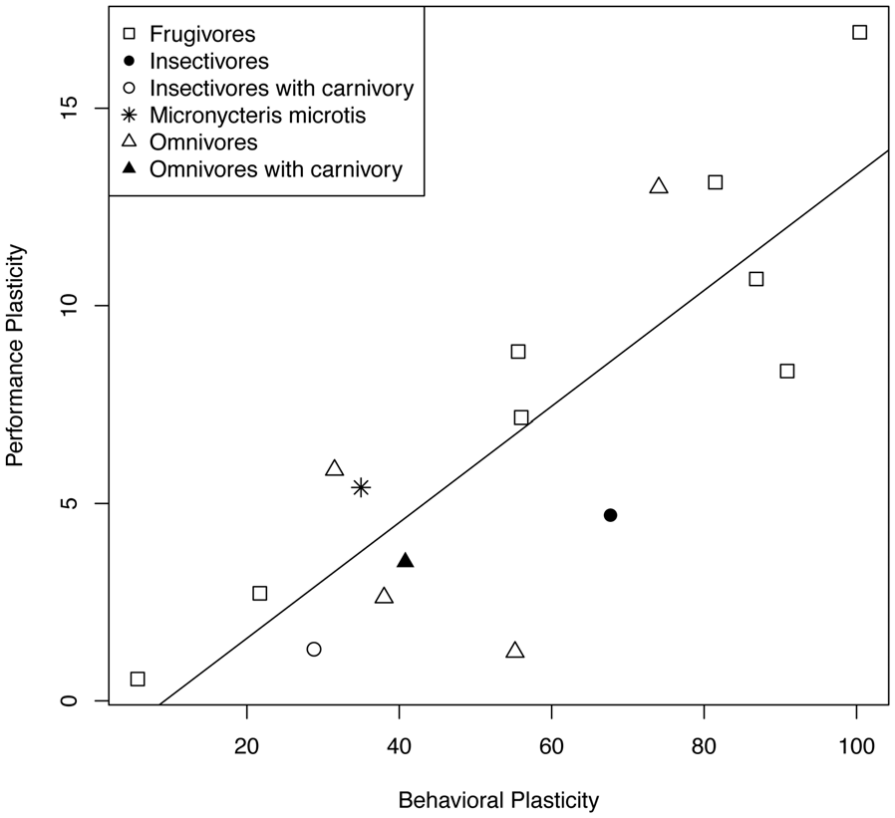
Behavioral and performance plasticity in phyllostomid bats while feeding on soft and hard prey items (fruits or insects). Species included for comparison are frugivores: Artibeus jamaicensis, Artibeus phaeotis, Carollia perspicillata, Centurio senex, Platyrrhinus helleri, Sphaeronycteris toxophyllum, Sturnira lilium and Uroderma bilobatum; insectivore: *Mimon crenulatum*; insectivore with carnivory (animalivory): *Tonatia saurophila*; omnivores: Carollia brevicauda, Glossophaga soricina, Phylloderma stenops, Phyllostomus discolor; omnivore with carnivory: *Phyllostomus hastatus*. (R^2^  =  0.684, F_1,14_  =  9.3679, P  =  7.738exp-05).

*M. microtis* also exhibited relatively high performance plasticity in relation to the other insectivorous phyllostomids in the dataset (Figure 4), although this plasticity did not stand out relative to the whole group of phyllostomids measured. The rate of evolution for performance plasticity was not significantly different from the rest of the phyllostomid lineage (t  =  0.371, df  =  14, P  =  0.358).

## Discussion

Within gleaning animalivores, *Micronycteris microtis* has an unusually broad diet, which not only spans many arthropod taxa, sizes and physical characteristics, but also includes vertebrate prey. Our goal was to investigate whether such a wide dietary breadth could be linked to morphological, behavioral and performance traits of the feeding apparatus. Cranial morphology and bite force appear to have evolved at a rapid rate in *M. microtis*, resulting in this species having the highest bite force among the species studied after accounting for cranial size and shape. Evolutionary increases in bite force can be achieved through changes in cranial shape that maximize mechanical advantage (i.e. the ratio of force output to muscle force), changes in muscle anatomy that result in higher force production (e.g. increases in mass, reduction of muscle fiber length), or both. At first glance, the skull of *M. microtis* does not appear to deviate from the generalized morphology of other animal-eating phyllostomids. However, a recent study on a closely related species, M. hirsuta, demonstrated that this bat has a skull that is stronger under feeding loads than two other insectivorous phyllostomids (*Lophostoma silvicolum* and *Tonatia saurophila*) [32]. M. hirsuta also has a higher mechanical advantage. Similar characteristics are likely shared by *M. microtis*, thus further research on its cranial anatomy and biomechanics is essential for a full understanding of the enhanced biting performance of this species.

*M. microtis* also exhibits behavioral attributes that set it apart from other gleaners. Like other animal-eating bats, *M. microtis* predominantly uses its molars and premolars to process food items, but it apparently has an increased ability to modify its bite performance behaviorally (i.e. high performance plasticity) when compared to sympatric species. Although further studies are required to test this trend statistically, this is an intriguing finding, as one might expect canalization and lower behavioral plasticity within the highly diverse assemblages to which *M. microtis* belongs. Elevated performance plasticity, along with an elevated bite force, may be key traits that allow *M. microtis* to expand its niche space by accessing prey with a wider range of sizes and hardnesses, including vertebrates. This hypothesis is further supported by our finding that sympatric gleaning bats with lower performance plasticity than *M. microtis* also seem to have narrower diets. For example, *Mimon crenulatum* feeds mostly on beetles and moths, and *Tonatia saurophila* on katydids and beetles [11]. It is important to note, however, that we cannot exclude biases regarding the completeness of dietary information until more extensive dietary studies are conducted on these species.

Our evidence based on morphological, performance and behavioral adaptations goes hand-in-hand with preliminary data on the prey detection strategies used by *M. microtis* [10].A series of behavioral experiments indicate that *M. microtis* has also evolved a unique sensory strategy to locate prey. This species is able to detect silent and motionless prey with echolocation alone [10], as opposed to using the multisensory strategy employed by other animal-eating bats [33], [34]. Interestingly, the general structure and pattern of echolocation calls of *M. microtis* does not differ from other similar-sized gleaners and other phyllostomids with different diets [35]. Moreover, *M. microtis* hunts on the wing, checking leaf by leaf in the forest while hovering up and down the understory vegetation [36]. We conclude that the high feeding performance, behavior and dietary plasticity described here, combined with sensory specialization, makes *M. microtis* unique among coexisting phyllostomid species and may set it apart from other gleaning bats.

Only 13 of the approximately 1,100 extant species of bats are known to feed on vertebrate prey, including the large (>>10 g) phyllostomid gleaners (*Phyllostomus hastatus,* *Trachops cirrhosus*, *Tonatia saurophila*, *Chrotopterus auritus* and *Vampyrum spectrum*) [16], [37], [38]. These carnivorous bats tend to have relatively large body sizes when compared to closely related species and to non-carnivorous bats. Given the small size of *M. microtis* (body mass: 5–7 g), it was surprising to document the consumption of vertebrate prey in this species. Although there is a strong historical component to diet within phyllostomid lineages [9], [39], [40], until now none of the small *Micronycteris* species has been reported to consume vertebrate prey. As the dietary data for other small *Micronycteris* is not as extensive as for *M. microtis*, we cannot exclude that consumption of small vertebrates might also occur in these bats. Nevertheless, our finding disproves the long-standing assumption that a large body size is a requirement for carnivory in bats, as long as the prey item still fits into the bat’s gape angle and can be successfully processed by its bite force.To date, no cranial characteristics separate carnivorous bats from species that specialize on soft insects [11], [41], [42]. Both groups have elongated faces, thin dentaries, low condyles, and large brain cases [42]. Apparently, marked changes in skull morphology and function are not required to transition from insectivory to carnivory in bats [11]. Our results lend some support to this hypothesis, given that *M. microtis* used similar prey processing behaviors while feeding on insects and vertebrates. Further investigation on the diets of other gleaning phyllostomids are highly likely to reveal more instances of opportunistic carnivory. Within this context, small species such as *M. microtis* illustrate the performance and behavioral characteristics that would connect insectivory with carnivory. These findings also illustrate a dietary continuum instead of the traditional separation between insectivory and carnivory in bats, and speaks for the use of the term “animalivorous” when referring to these species.

Our study highlights the importance of documenting diet, feeding performance and plasticity for a better understanding of resource use and partitioning among tropical species. As the threat of extinction increases for tropical bats due to habitat destruction, the attributes that allow of *M. microtis* to find and process a wide range of prey could also help it tolerate habitat fragmentation and isolation [43], [44]. Indeed, this species appears to be increasing in numbers on isolated islands in the Barro Colorado Nature Monument. Although this could be caused in part by a competitive release from other animalivorous gleaners, it also may be due to the high efficiency of this species in finding food on very small spatial scales and its ability to behaviorally adjust its already high bite performance to various prey types.

## References

[1] Erwin T (2002). Tropical forests: their richness in Coleoptera and other arthropod species.. Foundations of tropical forest biology: classic papers with commentaries.

[2] Gentry A (1988). Tree species richness of upper Amazonian forests.. Proc Natl Acad Sci U S A.

[3] Myers N, Mittermeier R, Mittermeier C, da Fonseca G, Kent J (2000). Biodiversity hotspots for conservation priorities.. Nature.

[4] Hutchinson GE (1959). Homage to Santa Rosalia or why are there so many kinds of animals?. Am Nat.

[5] Bumrungsri S, Leelapaibul W, Racey P (2007). Resource partitioning in sympatric *Cynopterus* bats in lowland tropical rain forest, Thailand.. Biotropica.

[6] Schoener T (1974). Resource partitioning in ecological communities.. Science.

[7] Wright J (2002). Plant diversity in tropical forests: a review of mechanisms of species coexistence.. Oecologia.

[8] Toft C (1980). Feeding ecology of thirteen syntopic species of anurans in a seasonal tropical environment.. Oecologia.

[9] Giannini NP, Kalko EKV (2004). Trophic structure in a large assemblage of phyllostomid bats in Panama.. Oikos.

[10] Geipel I (2007). Prey detection of the Neotropical leaf-nosed bat *Micronycteris microtis* in Panamá..

[11] Giannini NP, Kalko EKV (2005). The guild structure of animalivorous leaf-nosed bats of Barro Colorado Island, Panama, revisited.. Acta Chiropterol.

[12] Kalka M, Kalko EKV (2006). Gleaning bats as underestimated predators of herbivorous insects: diet of *Micronycteris microtis* (Phyllostomidae) in Panama.. J Trop Ecol.

[13] Strait SG (1993). Molar morphology and food texture among small-bodied insectivorous mammals.. J Mammal.

[14] Evans AR, Sanson GD (2005). Biomechanical properties of insects in relation to insectivory: cuticle thickness as an indicator of insect 'hardness' and 'intractability'.. Aust J Zool.

[15] Kalko EKV, Estrada Villegas S, Schmidt M, Wegmann M, Meyer CFJ (2008). Flying high: assessing the use of the aerosphere by bats.. Integ Comp Biol.

[16] Simmons NB, Wilson DE, Reeder DM (2004). Order Chiroptera.. Mammal species of the World: a taxonomic and geographic reference.

[17] Simmons NB, Voss RS (1998). The mammals of Paracou, French Guiana: A Neotropical lowland rainforest fauna..

[18] Schnitzler HU, Kalko EKV (2001). Echolocation by insect-eating bats.. Bioscience.

[19] Siemers BM, Schnitzler HU (2004). Echolocation signals reflect niche differentiation in five sympatric congeneric bat species.. Nature.

[20] Aguirre LF, Herrel A, Van Damme R, Matthysen E (2003). The implications of food hardness for diet in bats.. Funct Ecol.

[21] Santana SE, Dumont ER (2009). Connecting behaviour and performance: the evolution of biting behaviour and bite performance in bats.. J Evol Biol 22:.

[22] Dumont ER (1999). The effect of food hardness on feeding behaviour in frugivorous bats (Phyllostomidae): an experimental study.. J Zool.

[23] Herrel A, De Smet A, Aguirre LF, Aerts P (2008). Morphological and mechanical determinants of bite force in bats: do muscles matter?. J Exp Biol.

[24] Santana SE, Dumont ER, Davis JL (2010). Mechanics of bite force production and its relationship to diet in bats.. Funct Ecol.

[25] Santana SE, Strait S, Dumont ER (2011). The better to eat you with: functional correlates of tooth structure in bats.. http://dx.doi.org/10.1111/j.1365-2435.2011.01832.x.

[26] Herrel A, Spithoven L, Van Damme R, De Vree F (1999). Sexual dimorphism of head size in *Gallotia galloti*: testing the niche divergence hypothesis by functional analyses.. Funct Ecol.

[27] Dumont ER, Herrel A (2003). The effects of gape angle and bite point on bite force in bats.. J Exp Biol.

[28] Jungers WL, Falsetti AB, Wall CE (1995). Shape, relative size, and size adjustments in morphometrics.. Am J Phys Anthropol.

[29] McPeek MA (1995). Testing hypotheses about evolutionary change on single branches of a phylogeny using evolutionary contrasts.. Am Nat.

[30] Paradis E, Strimmer K, Claude J, Jobb G, Opgen-Rhein R (2006). APE: Analyses of Phylogenetics and Evolution.. R package version.

[31] Harmon L, Weir J, Brock C, Glor R, Challenger W (2008). GEIGER: investigating evolutionary radiations.. Bioinformatics.

[32] Santana SE, Dumont ER (2011). Do roost-excavating bats have stronger skulls?. Biol J Linn Soc.

[33] Kalko EKV, Schnitzler HU, Kunz TH, Racey, PA (1998). How echolocating bats approach and acquire food.. Bat biology and conservation.

[34] Tuttle MD, Ryan MJ (1981). Bat predation and the evolution of frog vocalizations in the Neotropics.. Science.

[35] Kalko E, Brigham M, Jones G, Kalko E (2004). Neotropical leaf-nosed bats (Phyllostomidae): "Whispering" bats or candidates for acoustic survey.. Austin: Bat Conservation International.

[36] van de Sand M (2004). Ökologie und Verhalten einer neotropischen Fledermausart: Aktivität, Raumnutzung und Beutefang von *Micronycteris microtis* in Panamá..

[37] Dondini G, Vergari S (2000). Carnivory in the greater noctule bat (*Nyctalus lasiopterus*) in Italy.. J Zool.

[38] Norberg U, Fenton M (1988). Carnivorous bats?. Biol J Linn Soc.

[39] Wetterer AL, Rocman MV, Simmons NB (2000). Phylogeny of phyllostomid bats (Mammalia: Chiroptera): data from diverse morphological systems, sex chromosomes and restriction sites..

[40] Rojas D, Vale Á, Ferrero V, Navarro L (2011). When did plants become important to leaf-nosed bats?. http://dx.doi.org/10.1111/j.1365-294X.2011.05082.x.

[41] Freeman PW (1979). Specialized insectivoy: beetle-eating and moth-eating molossid bats.. J Mammal.

[42] Freeman PW (1984). Functional cranial analysis of large animalivorous bats (Microchiroptera).. Biol J Linn Soc.

[43] Albrecht L, Meyer C, Kalko E (2007). Differential mobility in two small phyllostomid bats, *Artibeus watsoni and Micronycteris microtis*, in a fragmented Neotropical landscape.. Acta Theriol.

[44] Meyer C, Kalko E (2008). Assemblage level responses of phyllostomid bats to tropical forest fragmentation: land bridge islands as a model system.. J Biogeogr.

