# Correction: All You Can Eat: High Performance Capacity and Plasticity in the Common Big-Eared Bat, *Micronycteris microtis* (Chiroptera: Phyllostomidae)

**DOI:** 10.1371/journal.pone.0339697

**Published:** 2025-12-23

**Authors:** The PLOS One Staff

The last row of Table 1 has been removed. Please see the correct Table 1 here.

Shallow unilateral bites are not indicated as these were very rarely used. Sample sizes for hardness of prey items are as follows: beetles: 139; cicadas: 2; dragonflies: 1; katydids: 29; roaches: 2; stick insects (phasmids): 3. *M. microtis* maximum bite force is 8.25 ± 1.5 N.

The publisher apologizes for the error.

**Table 1:** Characteristics of the prey items and feeding behavior of *Micronycteris microtis.*

Prey type	N	Prey length (mm)	Prey width (mm)	Prey hardness (N)	% Shallow bilateral	% Deep unilateral	% Deep bilateral	Number of bites per prey item	Total time per prey item (sec)
Beetles	24	11.86 ± 2.61	4.79 ± 1.75	2.16 ± 0.68	0	70.62 ± 37.39	29.37 ± 37.39	791.24 ± 298.51	177.87 ± 87.11
Caterpillars	45	29.39 ± 9.54	4.13 ± 1.08	--	0	87.00 ± 25.36	10.78 ± 21.68	988.52 ± 560.02	220.40 ± 153.64
Cicadas	2	11.68 ± 0.96	4.25 ± 0.81	1.46 ± 0.42	0	59.17 ± 49.03	7.50 ± 18.37	289.00 ± 1.41	64.50 ± 0.70
Dragonflies	30	35.59 ± 5.42	2.36 ± 0.48	2.36	0	83.83 ± 31.72	9.50 ± 22.21	857.32 ± 354.83	179.30 ± 69.46
Hymenoptera	9	9.34 ± 1.64	1.20 ± 0.48	--	0	64.44 ± 48.76	24.44 ± 43.33	269.22 ± 34.76	59.00 ± 10.44
Katydids	45	17.49 ± 4.21	3.95 ± 0.92	1.89 ± 0.39	1.67 ± 11.18	84.22 ± 30.98	9.67 ± 22.79	1048.76 ± 696.36	241.67 ± 186.46
Lizards	1	39.25	3.36	--	0	100	0	980	210
Moths	30	17.71 ± 3.99	4.55 ± 1.52	--	0.17 ± 0.91	73.67 ± 32.90	26.17 ± 33.02	682.81 ± 462.32	155.50 ± 112.41
Neuropterans	1	40.13	1.87	--	0	100	0	544.29	127
Roaches	30	17.17 ± 6.39	3.10 ± 0.59	0.46 ± 0.335	0	79.17 ± 37.46	15.83 ± 28.58	1062.20 ± 1354.80	198.40 ± 241.34
Spiders	15	13.32 ± 2.53	3.41 ± 0.78	--	0	67.33 ± 42.58	32.67 ± 42.58	607.84 ± 216.43	146.60 ± 63.61
Stick insects	15	65.98 ± 14.79	3.16 ± 0.95	4.09 ± 1.79	0	63.00 ± 46.70	10.33 ± 26.01	991.10 ± 451.29	199.80 ± 67.23
